# Predicting Pathogenicity of *TSHR* Missense Variants of Uncertain Significance: An Integrative Computational Study

**DOI:** 10.3390/ijms27031614

**Published:** 2026-02-06

**Authors:** Tassneem Awad Hajali, Islamia Ibrahim Ahmed Omer, Mohamad Y. Rezk, Hamdan Z. Hamdan

**Affiliations:** 1 Department of Clinical Science, College of Medicine, Sulaiman AlRajhi University, P.O. Box 777, AlBukayriah 51941, Qassim, Saudi Arabia; t.hajali@sr.edu.sa (T.A.H.); i.omer@sr.edu.sa (I.I.A.O.); 2 Department of Physiology, College of Medicine, Qassim University, P.O. Box 6655, Buraidah 51452, Qassim, Saudi Arabia; myyosf@qu.edu.sa; 3 Department of Pathology, College of Medicine, Qassim University, P.O. Box 6655, Buraidah 51452, Qassim, Saudi Arabia

**Keywords:** thyroid dysfunction, congenital hypothyroidism, genetics, missense variants, uncertain significance

## Abstract

Pathogenic variants in the thyroid-stimulating hormone receptor gene (*TSHR*) contribute to a wide spectrum of thyroid dysfunctions, ranging from congenital hypothyroidism to thyrotropin resistance. With the advancement of bioinformatics algorithms for variant effect prediction, assessing the pathogenic potential of variants has become increasingly important. This study aimed to investigate the pathogenic effects of *TSHR* variants classified as variants of uncertain significance (VUSs) in the gnomAD v4.1.0 database. *TSHR* variants listed in gnomAD v4.1.0 were retrieved and filtered to select missense VUSs based on ClinVar classifications. Multiple bioinformatics tools were used to assess the secondary and three-dimensional structures of the *TSHR*, as well as protein stability, evolutionary conservation, and molecular dynamics simulations. A total of 2760 *TSHR* variants were found in gnomAD v4.1.0, including 75 frameshifts, 80 splice-sites, 265 in the 3′ and 5′ untranslated regions, 422 synonymous, 892 others, and 1026 missense variants. Among these, 68 missense VUSs were identified and selected for bioinformatics analysis. Three variants (p.Cys29Trp, p.Leu57Pro, and p.Phe97Ser) were consistently predicted to be pathogenic by all the bioinformatics tools used. All three variants were located within the leucin-rich repeat domain extracellular region of the *TSHR* and within a highly conserved region across species. Molecular dynamics simulations for mutant proteins (p.Cys29Trp, p.Leu57Pro, and p.Phe97Ser) reveal structural instability in comparison to the wild protein. Comprehensive bioinformatics analysis revealed that three *TSHR* missense VUSs exhibited pathogenic potential. These variants may contribute to thyroid dysfunction by affecting the receptor’s structural and signalling integrity.

## 1. Introduction

Thyroid-stimulating hormone receptor (*TSHR*) is mapped to the long arm of chromosome 14 (14q24−q31) and comprises 10 exons encoding a 764-amino-acid cell surface receptor thyroid-stimulating hormone (TSH) in the thyroid gland [1]. Structurally, *TSHR* has three domains: an extracellular domain, a transmembrane domain, and an intracellular C-terminal domain. The extracellular domain, spanning amino acids 22–279, mediates ligand interaction, recognition, and binding. It consists of a leucine-rich repeat (LRR) domain and a hinge region, which together create a concave shape that accommodates the ligand TSH [2].

*TSHR* belongs to the G-protein-coupled receptor superfamily. Upon binding to its ligand, it activates the cyclic adenosine monophosphate (cAMP) and inositol trisphosphate/calcium signalling pathways in the thyroid gland. These pathways regulate thyroid gland growth, cellular differentiation, and thyroid hormone secretion [3]. Variants in *TSHR* affect thyroid gland differentiation and development, leading to thyroid dysgenesis and dysfunction. Accordingly, a spectrum of clinical phenotypes ranging from severe congenital hypothyroidism to mild non-goitrous hyperthyrotropinemia (thyrotropin resistance), as well as cases with thyroid adenoma and carcinoma, has been reported [3,4,5]. Pathogenic *TSHR* (OMIM: 603372) variants have been reported since 1995 as either gain- or loss-of-function mutations [6]. However, the genotype–phenotype correlation remains uncertain, and several variants have been classified as variants of uncertain significance (VUSs), which may pose a diagnostic dilemma [7].

A missense variant is an alteration in the DNA coding region that leads to the substitution of an amino acid in a translated protein [8]. Recent advances in bioinformatics have led to algorithms that predict the pathogenicity of missense variants with up to 80% accuracy [9,10]. In the present study, we assessed the pathogenicity of *TSHR* missense VUSs identified in the gnomAD v4.1.0 database using bioinformatics tools that analyse the protein secondary structure, three-dimensional (3D) structures, and protein stability. The results generated provide preliminary findings that can help better characterise their impact on the structure and function of the *TSHR* protein and identify pathogenic variants that may be candidates for better personalised treatment.

## 2. Results

### 2.1. Variant Identification and Retrieval

**Figure 1:**
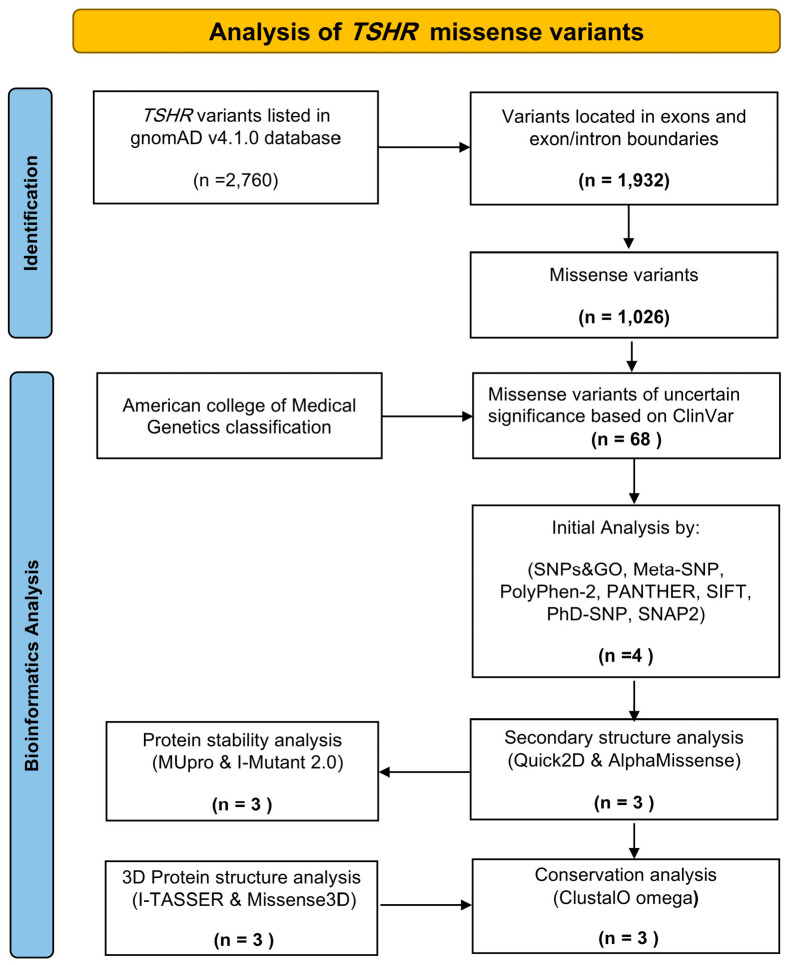
Methodological flowchart for the analysis of *TSHR* missense variants of uncertain significance.

After browsing the gnomAD v4.1.0 database, we identified a total of 2760 single-nucleotide polymorphisms (SNPs) associated with *TSHR*. Missense variants accounted for 37.1% of these SNPs (1026 variants), and 68 variants were flagged as having uncertain significance (see Table S1), which were selected for further bioinformatics analysis (see Figure 1).

### 2.2. Pathogenicity Assessment of Variants

To predict the potential pathogenicity of the VUSs, they were analysed using the following bioinformatics tools: Polymorphism Phenotyping v.2 (PolyPhen-2), SNPs & GO, Meta-SNP, Sorting Intolerant from Tolerant v.6.2.1 (SIFT), Protein Analysis Through Evolutionary Relationships v.19.0 (Panther), Predictor of Human Deleterious SNPs (PhD-SNP), and SNAP2 (v.1.0). Based on the analyses, only four of the 68 variants (p.Cys29Try, p.Leu57Pro, p.Gln90Pro, and p.Phe97Ser) were predicted to be pathogenic. The allele frequencies of the investigated variants are presented in Table 1. The most frequent variant was Leu57Pro, followed by Cys29Trp, Gln90Pro, and Phe97Ser.

### 2.3. Protein Stability Analysis

Protein stability analysis using I-Mutant 2.0 and MUpro 1.0 indicated that all four variants predicted to be pathogenic were predicted to have decreased protein stability (Table 2).

### 2.4. Secondary Structure Analysis

The four variants predicted to be pathogenic were selected for secondary structure analysis using two bioinformatics tools: AlphaMissense and Quick2D. The results of the Quick2D analysis revealed that all four variants exhibited structural dissimilarities with the native protein. The AlphaMissense results predicted three variants (p.Cys29Try, p.Leu57Pro, and p.Phe97Ser) as likely pathogenic and one variant (p.Gln90Pro) as likely neutral. Therefore, variant no. III was subsequently excluded from further analysis (see Table 3).

### 2.5. Conservation Analysis of the TSHR

**Figure 2:**
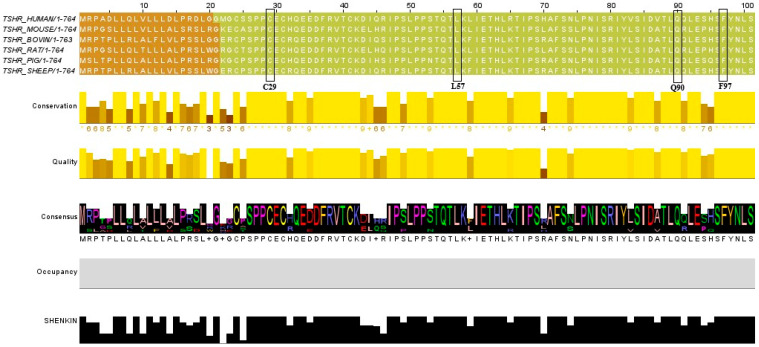
Multiple sequence alignment for *TSHR* across species. Black boxes indicate the position of the variants predicted to be pathogenic. All investigated variants were in positions that were highly conserved across the aligned species.

Multiple sequence alignment across several species (mouse, bovine, rat, pig, and sheep) revealed that the positions of all three variants predicted to be pathogenic by AlphaMissense are highly conserved among these species (Figure 2 and Table 3).

### 2.6. Results of Protein 3D Structure Prediction

**Figure 3:**
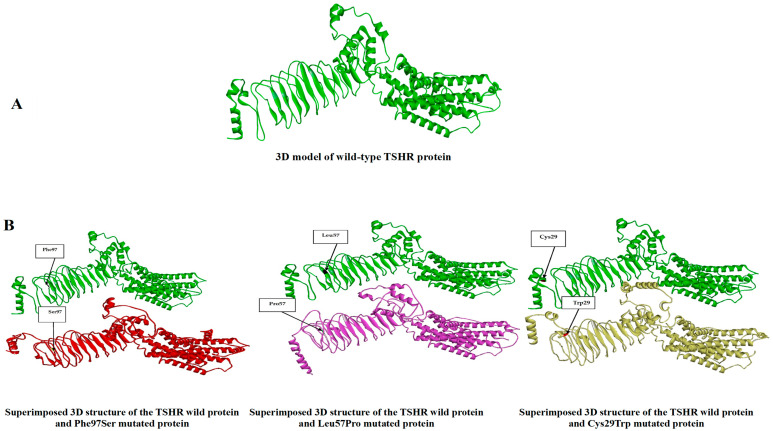
Three-dimensional protein structure analysis. (**A**) shows the 3D structure model of the *TSHR* wild protein. (**B**) Shows the superimposed wild protein structure model along with the mutated protein structure models for (Phe97Ser, Leu57Pro, and Cys29Trp).

The 3D structural analysis of the variants p.Cys29Try, p.Leu57Pro, and p.Phe97Ser using the Missense3D v.2.0 tool showed that all of them exhibited structural alterations and were predicted to be damaging (Table 4). Likewise, the Iterative Threading ASSEmbly Refinement (I-TASSER) server predicted the 3D structures of the wild and mutated forms of the *TSHR* protein, which were visualised using the BIOVIA Discovery Studio Visualizer v25.1.0.24284 (see Figure 3).

### 2.7. Protein–Protein Interaction Analysis

The Search Tool for the Retrieval of Interacting Genes/Proteins (STRING) database was used to analyse *TSHR*’s interaction network with other proteins. The 10 proteins with the highest binding scores are as follows (arranged from highest to lowest): TSHB, GNAS, TG, TPO, GNAQ, CGA, NKX2-1, IGF1R, FSHB, and SLC5A5 (see Figure S1).

### 2.8. Molecular Dynamics Simulations Analysis

**Figure 4:**
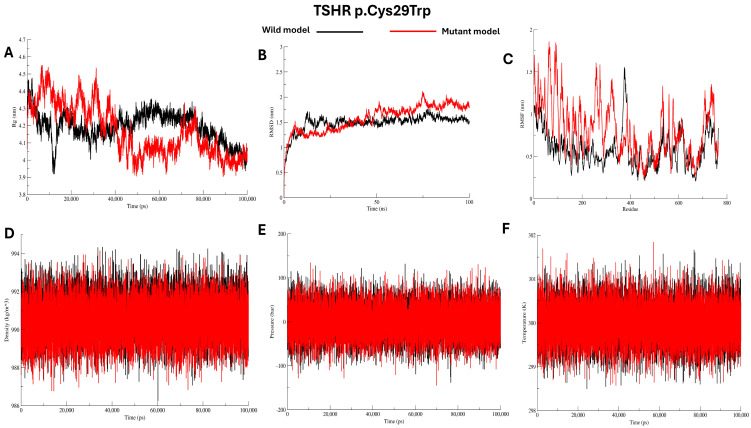
Analysis of wild *TSHR* model and p.Cys29Trp model using Gromacs molecular dynamics simulations (**A**–**F**).

**Figure 5:**
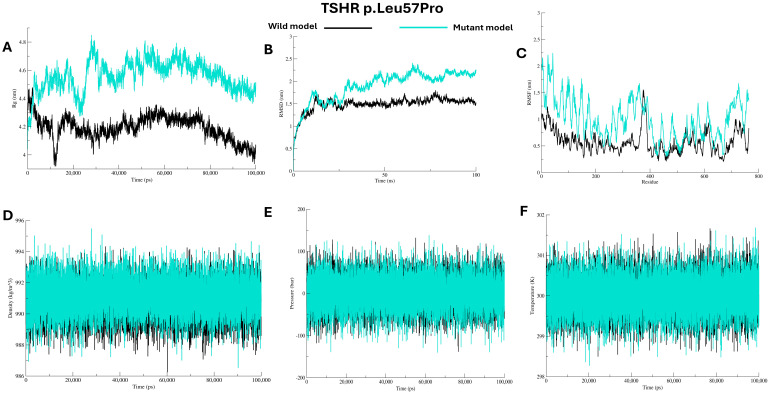
Analysis of wild *TSHR* model and p.Leu57Pro model using Gromacs molecular dynamics simulations (**A**–**F**).

**Figure 6:**
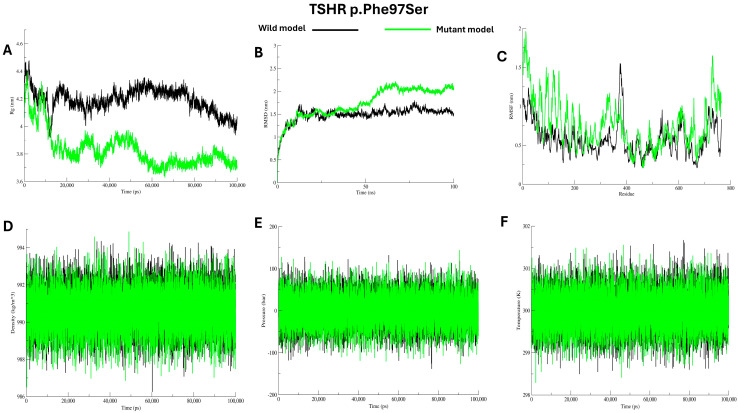
Analysis of wild *TSHR* model and p.Phe97Ser model using Gromacs molecular dynamics simulations (**A**–**F**).

Molecular dynamics (MD) simulations were performed to evaluate the microscopic stability, structural flexibility, and dynamic behaviour of the mutant proteins (Cys29Trp, Leu57Pro, and Phe97Ser) in comparison to the wild protein, see Figure 4, Figure 5 and Figure 6. Over the duration of 100 ns, the radius of gyration (Rg) for the wild protein is stable around 4.2–4.3 nm, indicating consistent structural packing. The mutant Cys29Trp model showed fluctuating Rg that reached ~4.5 nm during the first 10 ns, then steeply decreased to ~3.9 at 50 ns and rose to 4.2, then stayed at 4.0 until the end of the simulation, indicating abnormal folding, see Figure 4A. The mutant Leu57Pro showed the highest Rg value among the wild protein and other mutants, ranging from 4.6 to 4.8 nm, indicating abnormal folding and decreased protein stability, see Figure 5A. In contrast, mutant Phe97Ser showed the lowest Rg value, yet a fluctuating pattern between ~4.3 and ~3.7 nm, suggesting abnormal folding and a more compact structure, see Figure 6A.

Structural deviations were observed in the RMSD comparisons between the wild and mutant proteins. For the mutant Cys29Trp, a significant deviation is observed at 10 ns and 25 ns. At 50 ns, a substantial change in the entire trajectory was observed, as indicated in Figure 4B. Mutant Leu57Pro showed a deviation at 10 ns, and at 25 ns, the whole trajectory markedly deviated; see Figure 5B. For mutant Phe97Ser, multiple deviations were observed at 12 ns and 22 ns; at 30 ns, the trajectory markedly deviated and altered the entire track, see Figure 6B.

To investigate changes at the residue level, we calculated RMSF values comparing the wild protein to the mutant proteins. All mutant proteins showed markedly higher RMSF scores, especially around the positions of the substituted residues, see Figure 4C, Figure 5C, and Figure 6C (C). Other energy parameters, including temperature, pressure, and density, showed multiple minor and major deviations for all mutant proteins. See Figure 4, Figure 5 and Figure 6 (D–F).

## 3. Discussion

In the present study, bioinformatics tools were employed to identify missense VUSs in *TSHR*. Variants in *TSHR* may precipitate thyroid dysgenesis, a defect responsible for 85% of congenital hypothyroidism cases. Congenital hypothyroidism is considered sporadic; however, several reports indicate that familial forms—often inherited in autosomal dominant or recessive patterns—may account for up to 2% of cases [21,22]. Other clinical conditions, such as thyrotropin-resistant and non-autoimmune congenital hyperthyroidism, have also been attributed to *TSHR* variants [23,24].

The present study identified 68 *TSHR* missense variants classified as VUSs in gnomAD v4.1.0. Among these, three variants (p.Cys29Try, p.Leu57Pro, and p.Phe97Ser) were predicted by all the bioinformatics tools employed to have likely pathogenic effects. The first variant, c.87C>G (p.Cys29Try), is located within the first repeat of the LRR, which represents the extracellular domain of the receptor [3]. This LRR is organised spatially to form a horseshoe-shaped structure that facilitates protein–protein interactions [25]. Structurally, LRR comprises a well-organised complex right-handed β-α superhelix structure [26]. According to the Quick2D tool, the change from Cys29 to Try29 results in the loss of a nearby alpha-helix structure, imposing structural dissimilarity that may affect the binding affinity and function of *TSHR*. Additionally, Cys29 already forms a disulfide bridge with Cys24, which is essential for stabilising the tertiary structure of the protein [3]. A 3D structure analysis showed an altered 3D structure consistent with pathogenic potential. Moreover, size-wise, tryptophan is larger than cysteine, suggesting the introduction of a bubble structure on the protein surface. Physiochemically, tryptophan is a nonpolar amino acid, whereas cysteine is polar; such a difference can also distort the microenvironment of the protein pocket. Collectively, these findings may qualify this variant for consideration as a pathogenic variant.

Another missense variant that was predicted to be pathogenic in the present study was c.170T>C (p.Leu57Pro). This variant is also located within the LRR, precisely within the second repeat [3]. The introduction of a proline residue is known to disrupt the protein’s secondary structure because of its cyclic structure, which limits rotational movement around the α-carbon and α-amino bond. This substitution often results in kinks in α-helices or interruptions in β-sheets [27]. The Quick2D tool confirmed that p.Leu57Pro is associated with a loss of β-sheet structure, a main component of the LRR. Such loss of β-sheet may distort the LRR and interfere with ligand–receptor interaction.

Another finding in the present study was the pathogenic prediction of c.290T>C (p.Phe97Ser). This also belongs to the LRR of *TSHR*. Physiochemically, phenylalanine amino acid is nonpolar, while serine is polar; this difference may be associated with the loss of hydrophobic interaction within the protein core, which affects protein folding and stability [27]. Both the 3D and secondary structure analyses of *TSHR* revealed a structural dissimilarity of p.Phe97Ser with native *TSHR*, supporting its predicted pathogenicity.

The activation of *TSHR* involves a cascade of events initiated by the ligand binding to the extracellular domain. This initial interaction was thought to induce complex conformational changes in the hinge region and the transmembrane domain, which activate the G-protein to catalyse GTP hydrolysis and stimulate adenylate cyclase to produce cAMP, which then exerts its physiological role inside the target cell [28,29]. Variants located in the LRR were thought to decrease the constitutive activity of *TSHR* by 50% [30]. Perhaps amino acid substitutions in the LRR may interfere with this cascade and attenuate signal transduction. On the other hand, structural studies have indicated that some interactions between the extracellular domain and the transmembrane region are essential for maintaining receptor activity, and their loss through substitution variants is thought to increase the intrinsic activity of *TSHR* [31]. These two observations may provide a rationale for the diverse clinical phenotypes associated with *TSHR* LRR variants [32], highlighting the structural and functional significance of this domain. Of interest, conservation analysis of these variants showed that all three residues at Cys29, Leu57, and Phe97 are highly conserved across multiple species, emphasising their vital roles in the structure and function of the protein [33]. From an evolutionary perspective, conserved regions in a protein are preserved due to their indispensability for the protein function [34]. Hence, the Cys29, Leu57, and Phe97 residues are likely vital to *TSHR*’s structure and function.

The molecular dynamics simulation results for the variants predicted to be pathogenic revealed differences in radius of gyration and marked changes in RMSD. They increased RMSF scores, which align with the results of the bioinformatics tools—the radius of gyration reflecting the protein’s compactness and the stability of its 3D structure. In the present study, all mutant proteins showed different radii of gyration throughout the simulation, reflecting differences in folding and compactness compared to the wild protein structure. The RMSD analysis revealed that all mutant proteins exhibited multiple minor and major deviations along the trajectory. Larger deviation usually indicates a disturbed structure of the protein [35]. Collectively, we believe the MDS findings align well with the bioinformatics analysis results.

In the present study, we relied primarily on high-predictive-value bioinformatics tools. For instance, PolyPhen-2 is considered a baseline predictor, with 73–92% reported accuracy for predicting deleterious and damaging variants [11]. Likewise, SIFT can identify around 70% of disease-causing variants [14]. Moreover, SNP2 showed an area under the curve approaching 90.5% in predicting variants, which is above the 83.8% reported from SIFT and 85.3% reported from Polyphen-2 [17]. Capriotti et al. 2017 [36] reported 81% accuracy for SNPs&GO. Furthermore, a recent study by Tabet et al. 2024 [37], which employed 24 bioinformatics tools to predict pathogenicity variants in the UK Biobank, showed that each tool accurately identified more than 90% of disease-causing missense variants. SIFT, PolyPhen-2, and AlphaMissense were included in these sets. Although these bioinformatics tools provide valuable insights, none are error-free. Therefore, integrating multiple prediction algorithms with MDS analysis offers a more robust, scientifically sound approach to evaluating VUSs.

## 4. Materials and Methods

### 4.1. Variants Recruitment and Selection

We browsed the gnomAD database (version 4.1.0; https://gnomad.broadinstitute.org) and searched for *TSHR* variants. The complete list of *TSHR* variants was downloaded to the researcher’s computer using Microsoft Excel and then manually filtered in a stepwise process. First, only variants located within the coding region were selected. Second, missense variants were selected. Finally, variants categorised as VUSs based on the ClinVar classification were retained for further analysis.

### 4.2. Predicting Variants’ Pathogenicity

Bioinformatics tools [SNPs & GO (https://snps.biofold.org/snps-and-go/), Meta-SNP (https://snps.biofold.org/meta-snp/), Polymorphism Phenotyping v.2 (PolyPhen-2) (http://genetics.bwh.harvard.edu/pph2/), Protein Analysis Through Evolutionary Relationships (PANTHER v.19.0) PANTHER (https://pantherdb.org), Sorting Intolerant From Tolerant v.6.2.1 (SIFT) (https://sift.bii.a-star.edu.sg), Predictor of Human Deleterious SNPs (PhD-SNP) (https://snps.biofold.org/phd-snp/phd-snp.html), and SNAP2 v.1.0 (https://bio.tools/snap2)] were employed to predict pathogenic variants from neutral ones. Variants were sought in batches using the default settings of these tools.

### 4.3. Predicting Protein Secondary Structure

The secondary structure of the wild *TSHR* protein and the pathogenic variants identified by all the bioinformatics tools employed were predicted using Quick2D (https://toolkit.tuebingen.mpg.de/tools/quick2d) and AlphaMissense (https://www.science.org/doi/10.1126/science.adg7492).

### 4.4. Predicting Protein Stability

The protein stability of the variants predicted to be pathogenic by all the bioinformatics tools employed was evaluated using MUpro v.1.0 (http://mupro.proteomics.ics.uci.edu) and I-Mutant v.2.0. (https://folding.biofold.org/i-mutant/i-mutant2.0.html).

### 4.5. Predicting Protein 3D Structure

The 3D structures of the wild *TSHR* protein and selected pathogenic variants were modelled using the I-TASSER server (https://seq2fun.dcmb.med.umich.edu/I-TASSER/). Missense3D (https://missense3d.bc.ic.ac.uk/missense3d/) was subsequently employed to identify potential structural alterations in the 3D models resulting from amino acid substitutions. The BIOVIA Discovery Studio Visualizer v25.1.0.24284 software was used to visualise the 3D structure models.

### 4.6. Conservation Analysis

We used Jalview (version 2.11.5.0) to assess the degree of conservation of the *TSHR* protein across species. We obtained amino acid sequences for the human *TSHR* protein and its orthologs in other species from the NCBI HomoloGene database (https://www.ncbi.nlm.nih.gov). In Jalview, we performed multiple sequence alignments and checked for consensus sequences. These steps helped us to identify conserved residues among the species we studied.

### 4.7. Protein–Protein Interaction

The interaction network of *TSHR* with other proteins was analysed using STRING (https://string-db.org). This database integrates information from co-expression patterns, experimental evidence, functional associations, and gene fusion events to predict protein–protein interactions.

All the bioinformatics tools/websites used were accessed within the period from 3 September to 21 October 2025.

### 4.8. Molecular Dynamics Simulations

Pathogenic predicted variants were analysed using molecular dynamics (MD) simulations with GROMACS version 2025.1. Before simulation, protein systems were parameterized using the CHARMM36 force field. They were then energy-minimised to eliminate possible steric clashes. Each system was solvated in a cubic box with the SPC water model, under periodic boundary conditions. Charge neutrality was reached by adding Na^+^ and Cl^−^ ions. Two-step equilibration followed. First, a 100 ps NVT equilibration at 310 K was performed using the velocity-rescale thermostat. Next, a 100 ps NPT equilibration at 1 bar was carried out by the Parrinello–Rahman barostat. Positional restraints were applied to protein-heavy atoms. The LINCS algorithm constrained covalent bonds, and the Particle-Mesh Ewald method was used for long-range electrostatic interactions. Finally, two independent unrestrained production MD simulations of 100 ns each were conducted for all systems. Trajectories were recorded for subsequent analyses.

During the molecular dynamics (MD) simulations, a set of structural and dynamic analyses was performed to compare the mutant (MT) and wild-type (WT) proteins. For each system, root-mean-square deviation (RMSD), root-mean-square fluctuation (RMSF), and radius of gyration (Rg) were calculated throughout the simulation. In addition, key thermodynamic parameters, namely, the system’s temperature, pressure, and density, were recorded to ensure system stability.

## 5. Conclusions

To the best of our knowledge, the present study was the first to employ computational analysis to investigate missense *TSHR* variants flagged as VUSs. Among the 68 missense VUSs identified, three (p.Cys29Trp, p.Leu57Pro, and p.Phe97Ser) were predicted to be pathogenic through comprehensive computational analysis. Nevertheless, the results of the present study were based solely on the use of bioinformatics tools, and to our knowledge, no study has reported the aforementioned variants in a clinical context. Therefore, a phenotype–genotype correlation cannot be established. Additionally, functional assays, including receptor expression, ligand binding, and cAMP signalling, are warranted to validate our findings. Therefore, further study is needed.

## Figures and Tables

**Table 1:** List of *TSHR* variants commonly predicted to be pathogenic by all tools.

Variant Information					Allele Frequency						Polyphen-2 ^a^		SNPs and Go ^b^		MetaSNP ^c^		SIFT ^d^		Panther ^e^		PhD-SNP ^f^		SNAP2 ^g^	
S. No	Chr:bp	Alleles	AA	AA Cord	Overall	Eur	Afr	Ame	E. Asi	Mid	Pred	Prob	Pred	Prob	Pred	Score	Pred	Score	Pred	Pres Time	Pred	Score	Pred	Score
I	14:80955767	C/G	Cys/Trp	29	4.956 × 10^−6^	0.0	0.0	0.0	4.456 × 10^−5^	0.0	PD	1	D	0.86	D	0.92	D	0.02	D	0.99	D	0.87	D	0.77
II	14:80955850	T/C	Leu/Pro	57	4.957 × 10^−6^	5.932 × 10^−6^	0.0	0.0	0.0	0.0	PD	1	D	0.52	D	0.59	D	0.01	D	0.69	D	0.66	D	0.71
III	14:81068280	A/C	Gln/Pro	90	1.86 × 10^−6^	8.479 × 10^−7^	1.334 × 10^−5^	0.0	0.0	1.651 × 10^−4^	PD	0.99	D	0.83	D	0.81	D	0.03	D	0.53	D	0.89	D	0.70
IV	14:81068301	T/C	Phe/Ser	97	6.199 × 10^−7^	8.479 × 10^−7^	0.0	0.0	0.0	0.0	PD	1	D	0.84	D	0.83	D	0.00	D	0.92	D	0.90	D	0.76

Key: Chr: Chromosome; bp: Base pair; AA: Amino acid; AA coord: Amino acid coordinate; Eur: European (non-Finn); Afr: African; Ame: American; E. Asi: East Asian; Mid: Middle Eastern; Pred: Prediction; Prob: Probability; Pres: Preservation; and D: Damaging. ^a^ Polyphen-2 scores [0–1] predict variants as benign (<0.15), possibly damaging (0.16–0.85), or probably damaging (>0.85) [11]. ^b^ SNPs and Go scores [0–1] predict variant as disease-causing (>0.5) or predict as neutral (≤0.5) [12]. ^c^ MetaSNP scores [0–1] predict variant as disease (>0.5) or neutral (≤0.5) [13]. ^d^ SIFT scores range from 0 (damaging) to 1 (tolerated), with a cut-off value of 0.05 [14]. ^e^ Panther scores have a range of [0–1] and predict variant as diseased (>0.5) or neutral (≤0.5) [15]. ^f^ PhD-SNP scores have a range of [0–1] and predict variant as diseased (>0.5) or neutral (≤0.5) [16]. ^g^ SNAP2 Output normalised between 0 and 1 (if >0.5, mutation is predicted as diseased) [17].

**Table 2:** Predicted protein stability analysis with the variants in *TSHR*.

Variant Information				I-Mutant2.0 ^a^		MuPro ^b^	
S. No.	rsID	Aa/Aa	Aa Position	Stability	RI	Stability	Score
I	rs777166186	Cys/Trp	29	Decrease	4	Decrease	−0.81
II	rs200401152	Leu/Pro	57	Decrease	5	Decrease	−2.15
III	rs768151924	Gln/Pro	90	Decrease	2	Decrease	−1.08
IV	rs1384603967	Phe/Ser	97	Decrease	7	Decrease	−1.80

Key: Aa: Amino acid, and RI: Real ability index. ^a^ I-Mutant2.0 reports that a ΔΔG < 0 indicates a destabilising protein structure [18]. ^b^ MUPro reports a stability-change score between [−1 and +1], and scores < 0.0 indicate decreased stability [19].

**Table 3:** Secondary structure analysis for the variants in *TSHR*.

Variant Information			AlphaMissense ^a^		Quick2D	ClustalO Omega Conservation Analysis V1.2.4
S. No	rsID	Aa/Aa	Stability	RI	Prediction	Result
I	rs777166186	p.Cys29Trp	Likely pathogenic	0.87	Absent alpha helix	+++
II	rs200401152	p.Leu57Pro	Likely pathogenic	0.99	Affects beta sheet structure	+++
III	rs768151924	p.Gln90Pro	Likely neutral	0.48	The alpha helix is absent	+++
IV	rs1384603967	p.Phe97Ser	Likely pathogenic	0.98	Affects the beta sheet	+++

Key: Aa: Amino acid, RI: Real ability index, +++; no change in amino acid. ^a^ AlphaMissense reports a probability ranging between [0 and 1], and a probability ≥ 0.56 predicted a pathogenic protein [20].

**Table 4:** Predicted 3D protein analysis with the variants in *TSHR*.

Variant Information					3D Changes	Prediction
S. No	Chr:bp Cord	Alleles	AA	AA cord		
I	14:80955767	C/G	Cys/Trp	29	Disulfide breakage	Damaging
II	14: 8095585	T/C	Leu/Pro	57	Buried proline	Damaging
III	14:81068301	T/C	Phe/Ser	97	Cavity altered	Damaging

Key: Chr: Chromosome, bp: Base pair, AA: Amino acid, and AA coord: Amino acid coordinate.

## Data Availability

The original data presented in the study are openly available in [Genome Aggregation Database v4.1.0] at https://gnomad.broadinstitute.org/.

## Supplementary Materials

The following supporting information can be downloaded at: https://www.mdpi.com/article/10.3390/ijms27031614/s1.

## Abbreviations

**Abbreviation**	**Definition**
3D	Three-dimensional
cAMP	Cyclic adenosine monophosphate
Cys	Cysteine
GTP	Guanosine triphosphate
I-TASSER	Iterative Threading ASSEmbly Refinement
Leu	Leucine
LINCS	Library of Integrated Network-based Cellular Signatures
LRR	Leucine-rich repeat
MDS	Molecular dynamics simulation
Meta-SNP	Meta-predictor for the functional impact of non-synonymous SNPs
MT	Mutant
NVT	Constant number of particles, volume, and temperature (NVT ensemble)
PANTHER	Protein Analysis Through Evolutionary Relationships
PhD-SNP	Predictor of Human Deleterious Single-Nucleotide Polymorphisms
Phe	Phenylalanine
PolyPhen-2	Polymorphism Phenotyping v2
Rg	Radius of gyration
RMSD	Root-mean-square deviation
RMSF	Root-mean-square fluctuation
SIFT	Sorting Intolerant From Tolerant
SNAP2	Screening for Non-Acceptable Polymorphisms 2
SNPs	Single-nucleotide polymorphisms
SNPs&GO	Predictor of disease-associated mutations using Gene Ontology
SPC	Simple Point Charge (water model)
STRING	Search Tool for the Retrieval of Interacting Genes/Proteins
TSH	Thyroid-stimulating hormone
*TSHR*	Thyroid-stimulating hormone receptor
VUS	Variants of uncertain significance
WT	Wild-type
