# Supplementary material for: Predicting Pathogenicity of TSHR Missense Variants of Uncertain Significance: An Integrative Computational Study
Source: Int J Mol Sci. 2026 Feb 6;27(3):1614. doi: 10.3390/ijms27031614 (PMC12898700; doi:10.3390/ijms27031614)
Supplement: Supplementary file 1 [file ijms-27-01614-s001.zip › ijms-4117947-supplementary.pdf]

Predicting Pathogenicity of *TSHR* Missense Variants of Uncertain Significance: An Integrative Computational Study

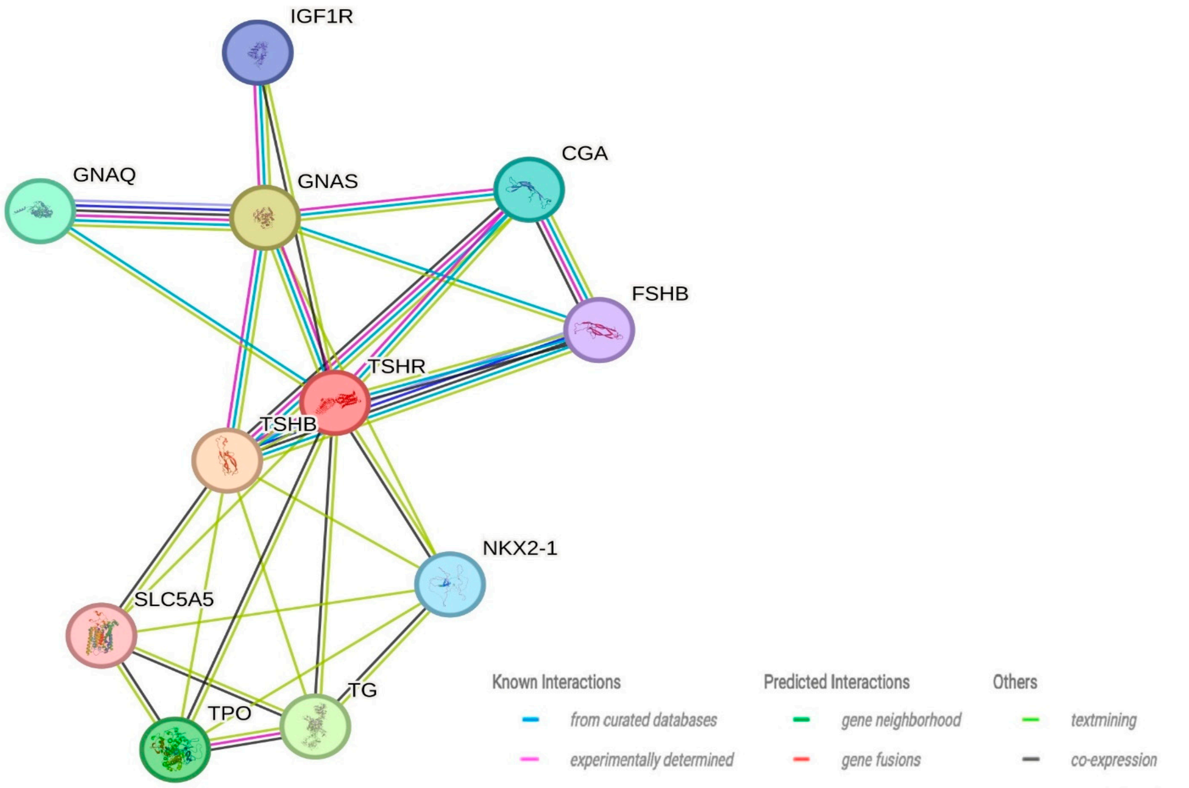

Figure S1. Protein-protein interaction network for TSHR. The network showed the top protein interacting with TSHR. Obtained from STRING v12.0.

Table S1. All missense variants with uncertain significance reported in gnomAD v.4.1.0

S.No	Chr:bp	rsID	Alleles	Protein consequence	Overall Allele frequency	Ancestry with maximum frequency	Allele frequency in the maximum ancestry
1.	14:80955693	rs755970622	G>A	p.Asp5Asn	2.23E-05	NFE	0.00002233
2.	14:80955767	rs777166186	C>G	p.Cys29Trp	4.96E-06	EAS	0.00000739
3.	14:80955774	rs769881177	C>T	p.His32Tyr	4.34E-06	EAS	5.79E-05
4.	14:80955837	rs886050853	A>C	p.Ser53Arg	7.43E-06	NFE	0.000005
5.	14:80955847	rs781625203	C>T	p.Thr56Ile	1.73E-05	SAS	5.14E-05
6.	14:80955850	rs200401152	T>C	p.Leu57Pro	4.96E-06	NFE	0.00000247
7.	14:81062174	rs1886292569	C>A	p.Thr66Asn	1.86E-06	AFR	0.00000443
8.	14:81068274	rs1279135603	C>T	p.Thr88Ile	3.72E-06	EAS	4.35E-05
9.	14:81068280	rs768151924	A>C	p.Gln90Pro	1.86E-06	NA	NA
10.	14:81068301	rs1384603967	T>C	p.Phe97Ser	6.20E-07	NA	NA
11.	14:81068328	NA	T>C	p.Ile106Thr	1.86E-06	NFE	2.80E-07
12.	14:81088008	rs146496347	G>T	p.Glu124Asp	4.59E-05	NFE	0.00004865
13.	14:81088015	rs1888418597	C>T	p.Leu127Phe	1.24E-06	NA	NA
14.	14:81091082	rs1888697548	A>G	p.Thr136Ala	6.20E-07	NA	NA
15.	14:81092543	rs748980987	C>A	p.Asp160Glu	6.82E-06	AMR	9.00E-05
16.	14:81092589	NA	T>C	p.Cys176Arg	1.24E-06	NA	NA
17.	14:81096691	rs559898532	A>G	p.Thr200Ala	1.12E-05	AFR	0.00014364
18.	14:81108404	rs145761776	T>C	p.Val215Ala	1.74E-05	SAS	0.00018083
19.	14:81108661	rs3783941	C>T	p.Arg248Cys	3.97E-05	NFE	0.00004019
20.	14:81139705	rs911354556	C>A	p.Ala240Asp	8.67E-06	NFE	0.00000615

21.	14:81139726	rs777223874	A>G	p.Glu247Gly	1.30E-05	AFR	0.00001746
22.	14:81139744	rs1891604207	T>C	p.Ile253Thr	1.24E-06	SAS	0.00000365
23.	14:81139762	rs1215944830	C>A	p.Thr259Asn	6.20E-07	NA	NA
24.	14:81139806	rs373712078	C>T	p.Arg274Trp	1.05E-05	AFR	0.00002549
25.	14:81142966	rs756847429	A>T	p.Glu303Val	3.72E-06	AFR	3.47E-05
26.	14:81142987	rs139286618	G>T	p.Arg310Leu	2.48E-06	AFR	0.00001744
27.	14:81143042	rs1231925448	G>T	p.Glu328Asp	4.34E-06	SAS	3.59E-05
28.	14:81143091	NA	G>T	p.Asp345Tyr	3.10E-06	NFE	1.24E-06
29.	14:81143122	rs1891769304	T>C	p.Phe355Ser	9.29E-06	NFE	0.00000763
30.	14:81143136	rs768336761	G>C	p.Glu360Gln	2.11E-05	NFE	0.00002074
31.	14:81143173	rs1204330294	A>C	p.Asn372Thr	3.59E-05	SAS	0.00030293
32.	14:81143194	NA	A>G	p.Gln379Arg	6.20E-07	NA	NA
33.	14:81143196	rs774993591	G>A	p.Ala380Thr	3.72E-06	NFE	7.90E-07
34.	14:81143227	rs371139156	G>T	p.Cys390Phe	3.35E-05	EAS	0.00002977
35.	14:81143247	NA	G>A	p.Val397Met	4.96E-06	NFE	0.00000292
36.	14:81143272	NA	T>C	p.Phe405Ser	6.20E-07	NA	NA
37.	14:81143280	rs199702292	T>C	p.Cys408Arg	7.43E-06	AMR	0.00000553
38.	14:81143286	rs121908868	G>A	p.Asp410Asn	7.43E-06	NFE	0.000005
39.	14:81143322	rs746029360	T>C	p.Trp422Arg	3.10E-06	AMR	0.00002255
40.	14:81143328	rs587778742	G>T	p.Val424Phe	8.05E-06	NFE	6.80E-07
41.	14:81143328	rs587778742	G>A	p.Val424Ile	1.18E-05	SAS	4.37E-05
42.	14:81143343	rs1271370001	C>G	p.Leu429Val	6.20E-07	NA	NA
43.	14:81143388	rs754118014	T>C	p.Tyr444His	2.48E-05	SAS	0.00022775
44.	14:81143469	rs925961475	G>A	p.Ala471Thr	1.43E-05	SAS	0.00002104
45.	14:81143481	rs760771267	C>T	p.Leu475Phe	1.86E-06	NFE	2.80E-07
46.	14:81143507	rs2140111192	C>A	p.Asn483Lys	6.20E-07	NA	NA
47.	14:81143512	rs1396470006	C>A	p.Ala485Asp	6.20E-07	NA	NA
48.	14:81143581	rs772490052	C>T	p.Ser508Leu	4.96E-06	SAS	0.00000875
49.	14:81143596	rs142814218	C>T	p.Thr513Met	5.58E-06	SAS	0.00000875

50.	14:81143598	rs759709895	G>A	p.Val514Ile	5.58E-06	AMR	7.73E-05
51.	14:81143605	rs765336502	C>A	p.Thr516Asn	1.74E-05	SAS	0.00021756
52.	14:81143617	rs754517766	G>C	p.Trp520Ser	1.61E-05	NFE	0.00001454
53.	14:81143638	rs1294870139	T>C	p.Met527Thr	1.24E-06	NA	NA
54.	14:81143641	rs571893270	G>A	p.Arg528His	2.85E-05	AMR	6.62E-05
55.	14:81143649	rs139892516	C>T	p.Arg531Trp	6.38E-05	NFE	0.00006393
56.	14:81143650	rs750198847	G>A	p.Arg531Gln	5.02E-05	AMR	6.62E-05
57.	14:81143670	rs151264748	G>T	p.Ala538Ser	1.92E-05	AFR	0.00025362
58.	14:81143689	rs1891805647	G>A	p.Gly544Glu	3.72E-06	NFE	1.24E-06
59.	14:81143701	rs1382824150	G>T	p.Cys548Phe	1.24E-06	NFE	2.80E-07
60.	14:81143715	rs121908872	G>T	p.Ala553Ser	3.72E-06	NFE	1.24E-06
61.	14:81143728	rs1237819854	T>G	p.Leu557Trp	2.48E-06	NFE	2.80E-07
62.	14:81143761	rs149978216	T>A	p.Ile568Asn	0.000102	NFE	0.00011209
63.	14:81143781	rs757875222	G>A	p.Glu575Lys	6.82E-06	AFR	0.00000443
64.	14:81143803	rs751266534	A>T	p.Tyr582Phe	1.36E-05	NFE	0.00001144
65.	14:81143862	rs577092617	G>A	p.Val602Met	1.36E-05	AFR	0.00017679
66.	14:81143896	rs540799629	A>G	p.Tyr613Cys	1.24E-05	EAS	0.00025854
67.	14:81143923	NA	T>C	p.Ile622Thr	6.19E-07	NA	NA
68.	14:81143955	rs28937584	G>A	p.Asp633Asn	7.43E-06	NFE	0.00000359

AFR: African; AMR: American; EAS: Eastern Asian; NA: Not available; NFE: Non-Finnish European; SAS: South Asian
